# GSK3*β* Exacerbates Myocardial Ischemia/Reperfusion Injury by Inhibiting Myc

**DOI:** 10.1155/2022/2588891

**Published:** 2022-04-29

**Authors:** Cong Wen, Meide Lan, Xin Tan, Xiaobo Wang, Zaiyong Zheng, Mingming Lv, Xuemei Zhao, Hao Luo, Yanxu Liu, Ping Wei, Rongchuan Yue, Houxiang Hu, Li Guo

**Affiliations:** ^1^Department of Cardiology, Affiliated Hospital of North Sichuan Medical College, Nanchong 637000, China; ^2^Cardiovascular Research Center, Affiliated Hospital of North Sichuan Medical College, Nanchong 637000, China; ^3^Department of Endocrinology, The Southwest Hospital of Third Military Medical University, Chongqing 400038, China; ^4^Academician Workstation, Affiliated Hospital of North Sichuan Medical College, Nanchong 637000, China

## Abstract

Myocardial ischemia/reperfusion (MI/R) injury is a life-threatening disease with high morbidity and mortality. Herein, the present study is conducted to explore the regulatory mechanism of GSK3*β* in MI/R injury regarding cardiomyocyte apoptosis and oxidative stress. The MI/R injury mouse model and hypoxic reoxygenation (H/R) cell model were established. The expression pattern of GSK3*β*, FTO, KLF5, and Myc was determined followed by their relation validation. Next, loss-of-function experiments were implemented to verify the effect of GSK3*β*/FTO/KLF5/Myc on cardiomyocyte apoptosis and oxidative stress in the MI/R injury mouse model and H/R cell model. High expression of GSK3*β* and low expression of FTO, KLF5, and Myc were observed in the MI/R injury mouse model and H/R cell model. GSK3*β* promoted phosphorylation of FTO and KLF5, thus increasing the ubiquitination degradation of FTO and KLF5. A decrease of FTO and KLF5 was able to downregulate Myc expression, resulting in enhanced cardiomyocyte apoptosis and oxidative stress. These data together supported the crucial role that GSK3*β* played in facilitating cardiomyocyte apoptosis and oxidative stress so as to accelerate MI/R injury, which highlights a promising therapeutic strategy against MI/R injury.

## 1. Introduction

Ischemic heart disease is the leading cause of global incidence and mortality. Timely restoration of reperfusion is currently the most effective treatment for myocardial infarction, but reperfusion will further lead to myocardial ischemia/reperfusion (MI/R) injury [1]. MI/R leads to increased oxidative stress [2], which further contributed to cardiomyocyte apoptosis [3]. Importantly, inhibition of oxidative stress and apoptosis exerts therapeutic significance in MI/R injury [4]. Thus, studying the underlying molecular mechanisms of oxidative stress and apoptosis in MI/R injury is critical for developing effective therapeutic strategies to attenuate MI/R injury.

Glycogen synthase kinase-3 (GSK3), probably the busiest kinase in most cells, is involved in many epidemic diseases, including cancers, inflammatory diseases, and mental and neurological diseases [5]. GSK-3*β*, one of the isoforms of GSK-3, a multifunctional and component-active kinase, is known to modulate multiple cellular processes [6]. The involvement of GSK3*β* in the development of MI/R injury has been widely documented [7–9]. Moreover, a previous study has indicated that GSK3*β* can inhibit the expression of FTO by phosphorylation [10]. FTO can catalyze the demethylation of mA residues in mRNA and regulate the cellular level of mA modification [11]. The RNA demethylase FTO possesses key potential in MI/R injury by modulation of mA demethylation [12]. In addition, Kruppel-like zinc-finger transcription factor 5 (KLF5), a member of the KLF family, participates in regulating the expression of a variety of genes so as to modulate a variety of cell functions [13]. Moreover, KLF5 is able to maintain cardiovascular remodeling which is closely related to the improvement of various ischemic diseases, including MI/R injury [14]. According to bioinformatics analysis in our study, it was revealed that FTO and KLF5 might regulate Myc expression. Myc, also known as c-Myc, is closely related to cell cycle progression in normal tissues and is able to regulate cell apoptosis [15]. Deletion of Myc has been substantiated to promote MI/R injury [16]. Based on previous studies, we hypothesized that GSK3*β* could potentially affect oxidative stress and cardiomyocyte apoptosis via the FTO/KLF5/Myc axis, which is implicated in the development of MI/R injury.

## 2. Methods

### 2.1. Ethics Statement

The experiments involving animals were performed in compliance with the recommendations in the *Guide for the Care and Use of Laboratory Animals* of the National Institutes of Health. All experimental procedures were implemented under the ratification of the Ethics Committee of Affiliated Hospital of North Sichuan Medical College (approval number: 202144).

### 2.2. Bioinformatics Analysis

The N6-methyladenosine- (m6A-) modified genes were obtained from the m6A2Target database. MI/R injury-associated expression profile GSE67308 (4 normal samples and 4 MI/R injury samples) was retrieved in the Gene Expression Omnibus (GEO) database followed by screening differentially expressed m6A-modified genes utilizing limma package in R language with *P* < 0.05 as the threshold. mRNA sequences and promoter sequences of downstream genes were obtained through the National Center for Biotechnology Information database and the UCSC, respectively. SRAMP was applied to predict the m6A sites of downstream genes and JASPAR database to predict the binding sites between transcription factors and downstream genes. The MEM database was adopted for coexpression analysis to verify the relationship among genes.

### 2.3. Establishment of Mouse Model of MI/R Injury

C57BL/6 mice (aged 8-10 weeks), GSK-3*β*^floxed/floxed^ mice, and Myosin6-Cre mice were purchased from The Jackson Laboratory (Bar Harbor, ME, USA) and kept in the specific-pathogen-free environment. The Myosin6-Cre mice were hybridized with GSK-3*β*^floxed/floxed^ mice to obtain the Cre-Floxed/+ mice as F1 generation. Then, mice at F1 generation were hybridized with each other to obtain the F2 generation of Cre-Floxed/Floxed homozygous mice which were fed with raloxifen (320 mg/kg/day) feed for 7 days. All mice except the control group were induced to MI/R injury. Mice were anesthetized with an isoflurane vaporizer. The left thoracic cavity was incised with a scalpel. The left anterior descending coronary artery was sutured with nylon sutures to induce ischemia, lasting for 30 min. After removal of the sutures, mice were subjected to reperfusion for 3 h (for Western blot analysis and oxidative stress determination) or 24 h (for determining cardiac function, apoptosis, and infarct size). After the end of MI/R, the chest cavities were closed and tracheal intubation was removed. The sham-operated mice had the same procedure except that the left anterior descending coronary artery was not sutured. After removing 8 mice that died during the model establishment, a total of 48 mice were included for the following experiments.

GSK3*β* cardiac-specific knockout mice (GSK3*β* CKO) were prepared, and those without GSK3*β* knockdown treatment were regarded as control (GSK3*β* WT). GSK3*β* CKO mice were then infected with adenovirus carrying short hairpin RNA (shRNA) against Myc (GSK3*β* CKO+shMyc). GSK3*β* CKO mice infected with adenovirus carrying shNC (GSK3*β* CKO+shNC) or GSK3*β* WT mice infected with adenovirus carrying shMyc (GSK3*β* WT+shMyc) were also used as control. There were 8 mice in each group.

Adenovirus (AAV9-shMyc) was purchased from Shanghai Genechem Co., Ltd. (Shanghai, China). The adenovirus was constructed using the company's GV119 vector. The sequence was designed according to the mouse Myc and completed by the company. Adenovirus-treated mice were anesthetized with an isoflurane vaporizer 7 days before MI/R induction and ventilated with an HX-300S animal ventilator (Chengdu Techman Software Co., Ltd., Chengdu, China). The pericardial sac was removed through a small incision in the left anterior chest. From the top of the left ventricle to the aortic root, 200 *μ*L of 2 × 10^11^ pfu/mL adenovirus was injected. At the same time, the aorta and pulmonary artery were cross-clamped, and the clamps were held for 20 s while the heart beat on a closed system. After removing the air and blood, the chest cavity was sutured. Mice were returned to the cage for recovery.

### 2.4. Echocardiography

After 24 h of MI/R, 1% sodium pentobarbital (P3761, Sigma-Aldrich, St. Louis, MO, USA) was injected intraperitoneally to anesthetize the mice. Mice were fixed on the wooden board. Color ultrasound diagnostic apparatus (Vevo 2100, Visualsonics, Toronto, Canada) was applied to measure the left ventricular end diastolic dimension (LVEDD), left ventricular end systolic diameter (LVESD), left ventricular ejection fraction (LVEF), left ventricular fraction shortening (LVFS), left ventricular systolic pressure (LVSP), and left ventricular end diastolic pressure (LVEDP).

### 2.5. Myocardial Enzyme Activity Detection

Peripheral blood of mice was collected 24 h after the MI/R. Levels of myocardial enzymes (MBs) including lactate dehydrogenase (LDH, A020-1-2, Nanjing Jiancheng Bioengineering Institute, Nanjing, China), creatine kinase (CK, A032-1, Nanjing Jiancheng Bioengineering Institute), CK-MB (H197, Nanjing Jiancheng Bioengineering Institute), brain natriuretic peptide (BNP; NBP2-70011, NOVUS, Colorado, USA), and cardiac troponin I (cTnI) (NBP3-00456, NOVUS) were detected using kits.

### 2.6. Hematoxylin and Eosin (HE) Staining

Mice were euthanized with an excess of sodium pentobarbital (100 mg/kg, intraperitoneal injection) 24 h after the MI/R. The whole myocardial tissue sections were selected, dried, fixed at ambient temperature (30 s), stained with hematoxylin at 60°C (60 s), treated with 1% hydrochloric acid alcohol differentiation solution (3 s), and stained with eosin (3 min). After dehydration with gradient alcohol (70%, 80%, and 95% ethanol and anhydrous ethanol), respectively, the sections were cleared with xylene (3 times per 5 min) and sealed with gum. The damage quantification of the corresponding ten regions was graded under a microscope (BX63, Olympus, Tokyo, Japan) using cell edema, apoptosis, necrotic cell proliferation, and bleeding based on a four-point system (0, histopathological changes ≤ 10%, 1 = 11-25%, 2 = 26-50%, 3 = 51-75%, and 4 = 76-100%) [17–19]. The average score was calculated and analyzed.

### 2.7. Triphenyltetrazolium Chloride (TTC) Staining

After 120 min of reperfusion, mice were euthanized followed by implementation of TTC staining for measuring infarct size [20]. The samples were photographed under the Sony camera. ImageJ software (National Institutes of Health, Maryland, USA) was adopted to calculate the area of myocardial infarction. The noninfarct area was the blue-stained area, the infarct area was the gray-white myocardium, and the red-stained area was the area at risk.

### 2.8. 2′,7′-Dichlorodihydrofluorescein Diacetate (DCFDA) Fluorescence

After 3 h of reperfusion, mice were euthanized. The whole myocardial tissues were selected for frozen sections which were incubated with 4 *μ*M DCFDA (D6470, Solarbio, Beijing, China) for 1 h (dark condition). Five different visual fields were captured under a confocal laser microscope (FV-1000/ES, Olympus, Tokyo, Japan) to observe and take pictures. Sections were quantified with the help of ImageJ software (National Institutes of Health, Maryland, USA).

### 2.9. Determination of Superoxide Dismutase (SOD) Activity and Malondialdehyde (MDA) Content

The SOD activity and MDA content in myocardial tissues and mouse cardiomyocytes were measured utilizing the Superoxide Dismutase Activity Assay kit (ab65354, Abcam, Cambridge, UK) and the Lipid Peroxidation (MDA) Assay kit (ab233471, Abcam, Cambridge, UK), respectively. Myocardial tissues were sampled after mice were reperfused for 3 h.

### 2.10. Culture and Transfection of Primary Mouse Cardiomyocytes

As previously reported, primary mouse cardiomyocytes were separated from the hearts of newborn C57/BL6 mice of 1-3 days old [21]. The cells at passage 1 were used for further experimentations. All cells were cultured in a 37°C incubator with 5% CO_2_.

Cells were transfected with short interfering RNAs (siRNAs) against GSK3*β*-1 (siGSK3*β*-1), siGSK3*β*-2, siFTO-1, siFTO-2, siKLF5-1, and their negative controls (NCs) (Supplementary Table 1). The abovementioned siRNA sequences were designed on the Life Technologies website and purchased by the Sangon Biotech company (Shanghai, China).

### 2.11. Cardiomyocyte Hypoxia/Reoxygenation Injury Model

Upon confluence, mouse cardiomyocytes reached about 80%, the normal medium was replaced with serum-free and sugar-free Dulbecco's modified Eagle medium (DMEM). Cardiomyocytes were placed in a hypoxic incubator with 5% CO_2_ and 95% N_2_ and incubated under hypoxia for 6 h. The medium was sucked up with a pipette and renewed with DMEM appended to sugar, 1% penicillin-streptomycin, 10% fetal bovine serum, and 4 mmol/L glutamine. Cardiomyocytes were cultured in a 37°C incubator with 95% air and 5% CO_2_ for 2 h.

### 2.12. Reactive Oxygen Species (ROS) Detection

For detecting ROS, cardiomyocytes were stained with MitoSOX Red (40778ES50, Yeasen, Shanghai, China, final concentration of 5 *μ*M) at 37°C for 15 min. After removal of MitoSOX Red solution, fresh cell culture medium preincubated at 37°C were added for further culturing. Five different visual fields were selected to observe and take pictures under a confocal laser microscope (FV-1000/ES, Olympus, Tokyo, Japan). ImageJ software (National Institutes of Health, Maryland, USA) was applied for quantification.

### 2.13. Reverse Transcription Quantitative Polymerase Chain Reaction (RT-qPCR)

For total RNA extraction, the TRIzol reagent (16096020, Invitrogen, CA, USA) was employed. RT was implemented by the use of the RT kit (RR047A, Takara, Tokyo, Japan) to generate cDNA. The RT-qPCR assay was operated utilizing PCR instrument (ABI7500, ABI, USA). All RT-qPCR experiments were set with 3 replicates. The primers (Supplementary Table 2) were designed on PubMed. The relative expression of target genes was measured utilizing the 2^-*ΔΔ*Ct^ method normalized to glyceraldehyde-3-phosphate dehydrogenase (GAPDH) (mRNA level).

### 2.14. Western Blot Analysis

Total protein from tissues and cells were extracted using radioimmunoprecipitation assay (RIPA) lysis buffer containing phenylmethylsulfonyl fluoride (PMSF), loaded onto a sodium dodecyl sulfate-polyacrylamide gel electrophoresis gel, and transferred electrophoretically to polyvinylidene fluoride membrane (1620177, Bio-Rad, Hercules, CA, USA). Following blockage utilizing 5% skimmed milk or 5% bovine serum albumin (BSA) for 1 h at ambient temperature, the membrane was probed with diluted primary antibodies to *β*-actin (4970, CST, MA, USA), GSK3*β* (ab32391, Abcam), FTO (ab94482, Abcam), phosphoserine (ab9332, Abcam), KLF5 (ab137676, Abcam), and c-Myc (18583, CST, MA, USA) overnight at 4°C. The membrane was reprobed with the secondary goat anti-rabbit (ab6721, Abcam) or anti-mouse (ab6789, Abcam) immunoglobulin G (IgG) antibody labeled by horseradish peroxidase (HRP) for 1 h at ambient temperature. The membrane was immersed in enhanced chemiluminescence reaction solution (1705062, Bio-Rad, Hercules, USA) at ambient temperature for 1 min and imaged on the Image Quant LAS 4000C gel imager (GE, NY, USA). The relative gray-scale ratio of the target protein to *β*-actin was calculated.

### 2.15. Immunohistochemistry

Mouse hearts were embedded in the paraffin and sliced with an ultrathin microtome. Sections were then deparaffinized with xylene, rehydrated with graded alcohol, and reacted with 3% hydrogen peroxide to block endogenous peroxidase activity. The sections were boiled in 10 mM sodium citrate (pH 6.0) (30 min), sealed in 10% normal goat serum (15 min), and probed with the antibodies to FTO (ab92821, Abcam), KLF5 (51586, CST, MA, USA), or c-Myc (18583, CST, MA, USA) overnight in a wet room at 4°C. The next day, the sections were incubated with the secondary antibody for 1 h at ambient temperature. The diaminobenzidine (DAB) kit (Invitrogen, CA, USA) was utilized for immunoreactivity testing.

### 2.16. Flow Cytometry Assay

Mouse cardiomyocytes were trypsinized into a single cell suspension with a concentration of 10^6^ cells/mL. Cell suspension was stained with 10 *μ*mol/L JC-1 (T4069, Sigma-Aldrich, St. Louis, MO, USA) at 37°C (10 min, dark condition). Cell suspension was resuspended in phosphate-buffered saline (PBS), followed by analysis with the FACS Aria flow cytometer (BD Biosciences, CA, USA).

### 2.17. RNA Immunoprecipitation (RIP)

The RIP kit (17-701, Millipore, Billerica, MA, USA) was operated for the RIP experiment [22]. The antibodies used in the RIP experiment include FTO (27226-1-AP, Proteintech, Wuhan, China) or m6A antibody (202003, Synaptic Systems, Germany), with anti-rabbit IgG (2729, CST) as a negative control. Myc mRNA primer sequence was F: CTGGATTTTTTTCGGGTAGT; R: TTACGCACAAGAGTTCCGTAGC.

### 2.18. Terminal Deoxynucleotidyl Transferase- (TDT-) Mediated dUTP-Biotin Nick End-Labeling (TUNEL) Staining

For tissues, the TUNEL kit (C1098, Beyotime) was applied. Mouse heart paraffin sections were deparaffinized with xylene for 5-10 min, deparaffinized again with fresh xylene for 5-10 min, and treated with ethanol for 5 min, 90% ethanol for 2 min, 70% ethanol for 2 min, and distilled water for 2 min. Sections were treated with 20 *μ*g/mL DNase-free proteinase K (ST532, Beyotime) at 20-37°C (15-30 min) and incubated in a 3% hydrogen peroxide solution prepared in PBS for 20 min at ambient temperature and in the biotin-labeled solution at 37°C (60 min, dark condition). Then, sections were cultured with the labeled reaction termination solution (10 min), incubated in 50 *μ*L streptavidin-HRP solution, and reacted with 0.2-0.5 mL DAB at ambient temperature (5-30 min). Sections were subsequently sealed, observed, and photographed under an inverted microscope.

For cells, the red fluorescent TUNEL kit (C1089, Beyotime) was employed for apoptosis detection. Cells were fixed with 4% paraformaldehyde for 30 min, reacted with 0.3% Triton X-100 for 5 min at ambient temperature, and incubated with 50 *μ*L TUNEL solution at 37°C (60 min, dark condition). After mounting with antifluorescence quenching solution, five different visual fields were selected under a confocal laser microscope (FV-1000/ES, Olympus) for observation and photographing.

### 2.19. Caspase-3 Activity Detection

The Caspase-3 activity detection kit (BC3830, Solarbio) was employed for testing the Caspase-3 activity [23]. The absorption wavelength was measured utilizing a microplate reader at 450 nm.

### 2.20. Chromatin Immunoprecipitation (ChIP)

ChIP was operated utilizing the EZ-Magna ChIP TMA kit (Millipore) as per instructions [24]. Logarithmically growing cardiomyocytes were cross-linked, sonicated (200~1000 bp chromatin fragment), and centrifuged at 14000 g for 10 min at 4°C. The collected supernatant (100 *μ*L, DNA fragment) was reacted in 900 *μ*L of ChIP Dilution Buffer, 20 *μ*L of 50x PIC, and 60 *μ*L of Protein A Agarose/Salmon Sperm DNA. About 20 *μ*L of supernatant was employed as input. The cross-linked chromatin samples were immunoprecipitated with 1 *μ*L of anti-rabbit KLF5 (51586, CST, MA, USA) or 1 *μ*L of anti-rabbit IgG (ab172730, Abcam, control). Immunoprecipitated DNA was analyzed by qPCR to detect whether the KLF5 antibody-enriched chromatin fragment contained Myc promoter sequence (F: 5′-CTATCACTCCACACACT-3′, R: 5′-GTACTCCGGCTCCGGGGT-3′).

### 2.21. Dual-Luciferase Reporter Gene Assay

The full-length dual-luciferase reporter gene plasmid containing the Myc promoter and the dual-luciferase reporter gene plasmid with deletion of KLF5 binding site were constructed, respectively. The above constructed plasmids and pcDNA3.1-KLF5 plasmid or NC were cotransfected into cardiomyocytes, respectively. Transfected cardiomyocytes were lysed 48 h after transfection and centrifuged at 12000 g for 1 min. The supernatant was collected. Luciferase activity was measured using the Dual-Luciferase® Reporter Assay System (Dual-Luciferase® Reporter Assay System, E1910, Promega, Madison, WI, USA). The luciferase activity was directly measured by the ratio of firefly luciferase activity to renilla luciferase activity.

### 2.22. Statistical Analysis

Measurement data processed by the use of SPSS 21.0 statistical software (IBM Corp. Armonk, NY, USA) were described as the mean ± standard deviation. Data showing normal distribution and homogeneity of variance were compared utilizing the unpaired *t*-test (between two groups) or one-way analysis of variance (ANOVA) with Tukey's post hoc test or repeated measurement ANOVA (among multiple groups). Data at different time points were compared by repeated measure ANOVA, followed by the Bonferroni post hoc test. *P* < 0.05 was indicative of statistical significance.

## 3. Results

### 3.1. High Expression of GSK3*β* in a Mouse Model of MI/R Injury

**Figure 1:**
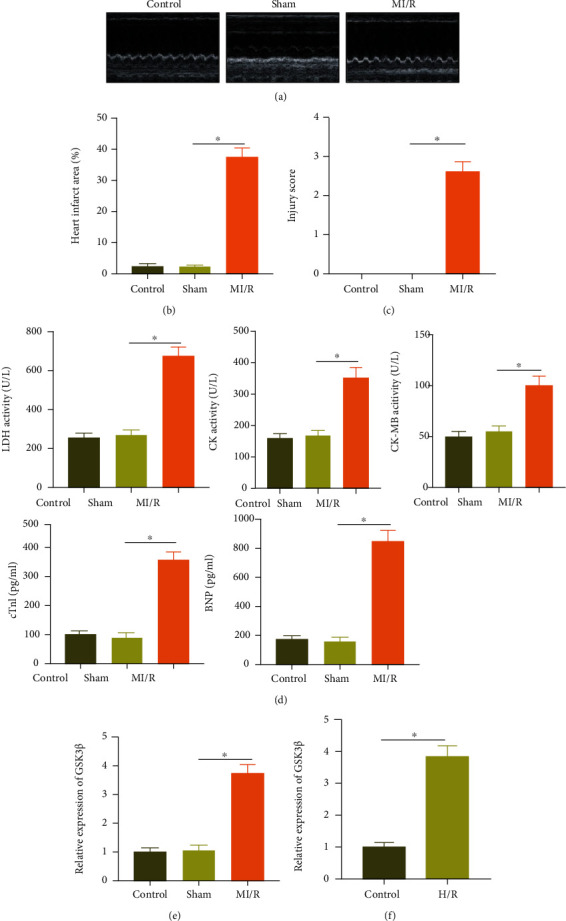
GSK3*β* shows high expression in the myocardial tissues of a mouse model of MI/R injury and H/R cell models: (a) M-mode echocardiogram images of mice in each group; (b) TTC staining statistics of myocardial tissue sections of mice in each group; (c) HE staining of myocardial tissue of mice in each group; (d) statistical graphs of the expression of LDH, CK, CK-MB, cTnI, and BNP in the peripheral blood of each group of mice; (e) RT-qPCR was applied to measure the expression of GSK3*β* in the myocardial tissue of each group of mice; (f) expression of GSK3*β* in the H/R-induced cardiomyocytes determined by RT-PCR. *N* = 8. Measurement data were expressed as the mean ± standard deviation. Data in compliance with normal distribution and homogeneity of variance between two groups were compared using the unpaired *t*-test. Comparisons among multiple groups were conducted by one-way ANOVA with Tukey's post hoc test. *p* < 0.05 was indicative of statistical significance. Cell experiment was repeated three times.

GSK3*β*, a protein with constitutive serine/threonine kinase activity, exerts a crucial function in modulating cell and tissue metabolism and is known as a hormone control regulator that maintained glucose stability [25]. At the same time, GSK3*β* could aggravate the MI/R [26]. For exploring the molecular mechanism of MI/R injury, we constructed a mouse model of MI/R injury. As shown by echocardiography, LVEDD, LVESD, and LVEDP were increased in the MI/R mice, and LVEF, LVFS, and LVSP were reduced (Figure 1(a), Supplementary Table 3). TTC staining showed that the area of myocardial infarction in the MI/R group was significantly increased (Figure 1(b)). Observation of HE staining showed that the cardiomyocytes in the control mice and the sham-operated mice were regular and arranged, with complete muscle fibers, and no cell edema or necrosis was observed. In the MI/R mice, some cardiomyocytes showed edema and necrosis, accompanied by local hemorrhage, neutrophil infiltration, and elevated pathological scores (Figure 1(c), Supplementary Figure 1A). The peripheral blood of each group of mice was taken to detect the expression of myocardial enzymes LDH, CK, CK-MB, cTnI, and BNP. The results showed that MI/R mice had elevated expression of LDH, CK, CK-MB, cTnI, and BNP (Figure 1(d)). These results suggested that the mouse model was successfully constructed.

RT-qPCR determination showed elevated GSK3*β* expression in the MI/R mice (Figure 1(e)). Then, we simulated MI/R injury at the cellular level and established a hypoxia-reoxygenation (H/R) model [1]. As demonstrated by RT-qPCR, an enhancement in GSK3*β* expression was also seen in the H/R-induced cells (Figure 1(f)).

These results suggested that GSK3*β* was highly expressed under MI/R injury.

### 3.2. Silencing GSK3*β* Inhibits MI/R Injury

**Figure 2:**
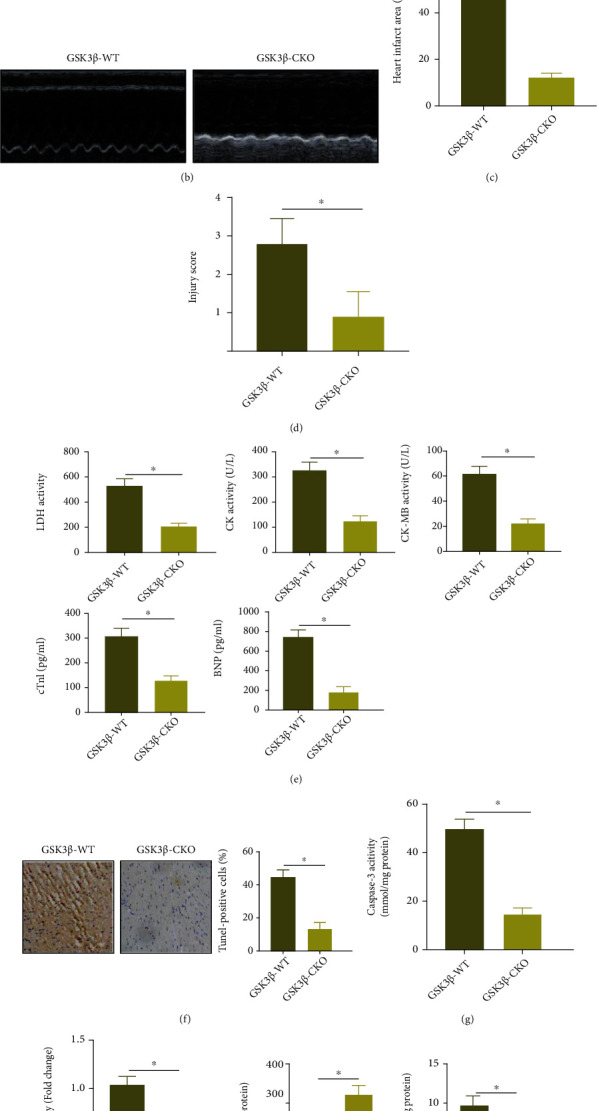
Deletion of GSK3*β* inhibits MI/R injury: (a) Western blot analysis was used to detect the protein expression of GSK3*β* in the myocardial tissue of GSK3*β* WT mice and GSK3*β* CKO mice; (b) M-mode echocardiogram images of GSK3*β* WT mice and GSK3*β* CKO mice; (c) TTC staining statistics of myocardial tissue sections of GSK3*β* WT mice and GSK3*β* CKO mice; (d) HE staining of myocardial tissue of GSK3*β* WT mice and GSK3*β* CKO mice; (e) statistics of the expression of LDH, CK, CK-MB, cTnI, and BNP in the peripheral blood of GSK3*β* WT mice and GSK3*β* CKO mice; (f) TUNEL detected the apoptosis of cardiomyocytes in GSK3*β* WT mice and GSK3*β* CKO mice; the scale bar was 25 *μ*m; (g) detection of Caspase-3 activity in the myocardial tissue of GSK3*β* WT mice and GSK3*β* CKO mice; (h) DCF-DA staining of the myocardial tissue section of GSK3*β* WT mice and GSK3*β* CKO mice to detect the expression of ROS in the cells; (i) the activity of SOD and the content of MDA in the hearts of GSK3*β* WT mice and GSK3*β* CKO mice. *N* = 8. Measurement data were expressed as the mean ± standard deviation. Data in compliance with normal distribution and homogeneity of variance between two groups were compared using unpaired *t*-test.

To explore the specific functions of GSK3*β* in MI/R injury, GSK3*β* CKO mice were constructed, in which GSK3*β* expression was extremely low (Figure 2(a)). After 40 min of ischemia and 24 h of reperfusion, cardiac echocardiography was started with the results depicting that compared with the GSK3*β* WT mice, LVEDD, LVESD, and LVEDP were decreased, but LVEF, LVFS, and LVSP were increased in the GSK3*β* CKO mice (Figure 2(b), Supplementary Table 4). TTC and HE staining noted that compared with the GSK3*β* WT mice, GSK3*β* CKO mice showed a reduced myocardial infarction area, alleviated pathological changes in myocardial tissues, and decreased infiltration of necrotic cardiomyocytes and centrifugal cells (Figures 2(c) and 2(d), Supplementary Figure 1B). Then, it was noted that the expression of LDH, CK, CK-MB, cTnI, and BNP in the GSK3*β* CKO mice was reduced (Figure 2(e)). These results suggested that silencing GSK3*β* alleviated MI/R injury.

Next, the TUNEL experiment demonstrated that relative to the GSK3*β* WT mice, the cardiomyocyte apoptosis in the GSK3*β* CKO mice was reduced (Figure 2(f)). Similarly, compared with the GSK3*β* WT mice, the Caspase-3 activity was limited in the GSK3*β* CKO mice (Figure 2(g)). These results suggested that silencing GSK3*β* suppressed MI/R injury-induced cardiomyocyte apoptosis.

The formation of ROS and oxidative stress are the key mechanisms of cell damage and dysfunction in cardiac MI/R injury [27]. For exploring the effect of GSK3*β* on oxidative stress, we used the ROS fluorescent probe DCFH-DA to stain the myocardial tissue sections of mice in each group. The results showed that ROS in the GSK3*β* CKO mice was significantly lower than that in the GSK3*β* WT mice (Figure 2(h)). In addition, in the GSK3*β* CKO mice, the activity of SOD increased while the content of MDA decreased (Figure 2(i)).

These results suggested that downregulation of GSK3*β* limited the cardiomyocyte apoptosis and oxidative stress caused by MI/R injury, thereby alleviating MI/R injury.

### 3.3. GSK3*β* Promotes Ubiquitination and Degradation of FTO and KLF5

**Figure 3:**
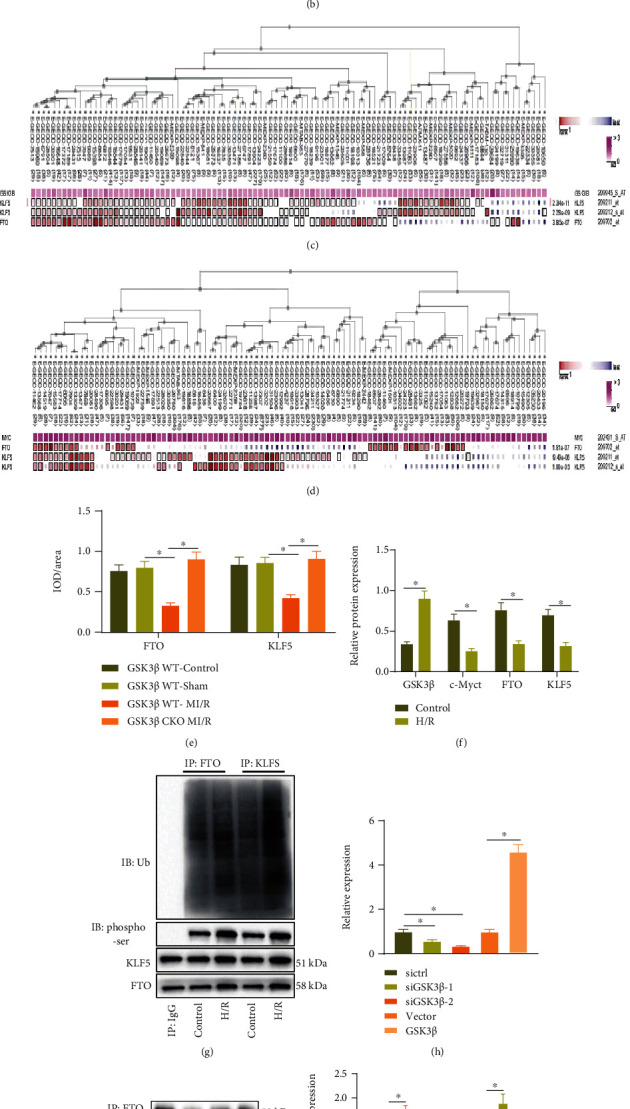
GSK3*β* phosphorylates FTO and KLF5 to promote their ubiquitination degradation. (a) The differential expression volcano map of m6A-modified genes in the microarray GSE67308; the red dots indicate upregulated genes, the green dots indicate downregulated genes, and the black dots indicate genes with insignificant differences. (b) The box plot of the expression data of FTO in the microarray GSE67308; the blue box on the left represents the expression of normal samples, and the red box on the right represents the expression of MI/R injury samples. (c) MEM analysis for the relation between GSK3*β* with FTO and KLF5. (d) MEM analysis for the relation between Myc with FTO and KLF5. (e) Immunohistochemistry was applied to detect the expression of FTO and KLF5 in the myocardial tissue of each group of mice. (f) Western blot analysis was used to determine the expression of FTO and KLF5 in the cardiomyocytes of each group. (g) The degree of ubiquitination and phosphorylation of FTO and KLF5 in the cardiomyocytes of each group detected by IP. (h) mRNA expression of GSK3*β* in the cardiomyocytes of each group detected by RT-qPCR. (i) IP detection of the phosphorylation degree of FTO and KLF5 in the cardiomyocytes of each group. (j) Western blot analysis detection of the expression of FTO and KLF5 in the cardiomyocytes of each group. (k, l) The corresponding plasmid was transfected into cardiomyocytes, 10 *μ*M MG132 was added after 36 h, the sample was collected after 8 h culture, FTO antibody (k) or KLF5 antibody (l) was used for immunoprecipitation experiment, and Ub antibody was used to detect ubiquitination level. (m) The corresponding plasmid was transfected into cardiomyocytes, CHX (50 *μ*g/mL) was added after 48 h, and samples were collected at 0 h, 3 h, 6 h, and 9 h after administration to detect the protein levels of FTO and KLF5. *N* = 8. Measurement data were expressed as the mean ± standard deviation. Data in compliance with normal distribution and homogeneity of variance between two groups were compared using unpaired *t*-test. Comparisons among multiple groups were conducted by one-way ANOVA or repeated measurement ANOVA with Tukey's post hoc test. Statistical analysis in relation to time-based measurements within each group was realized using repeated measurement ANOVA, followed by Bonferroni's post hoc test for multiple comparisons. *p* < 0.05 was indicative of statistical significance.

It has been reported that m6A modification can affect MI/R injury [28, 29]. We obtained 22 m6A modifier genes from m6A2Target and obtained 8 significantly different m6A modifier genes by analyzing the MI/R injury-related GSE67308 (Figure 3(a)). Among them, FTO was poorly expressed in MI/R injury, and the difference was the most significant (Figure 3(b)). Study shows that GSK3*β* accelerates its ubiquitination degradation through phosphorylation of FTO (phosphorylation sites S249 and S253) and KLF5 (human site S292 and mouse site S292) [10, 30]. Overexpression of FTO and KLF5 alleviated ischemia-reperfusion injury [12, 14]. Through MEM analysis, it was found that both FTO and KLF5 were significantly coexpressed with Myc, and GSK3*β* has a significant coexpression relationship with FTO and KLF5 (Figures 3(c) and 3(d)). Thus, we speculated that GSK3*β* may act as the upstream of FTO and KLF5 to regulate Myc expression and affect MI/R injury.

We then explored whether GSK3*β* regulated the expression of FTO and KLF5 in MI/R injury. As reflected by immunohistochemistry, compared with GSK3*β* WT sham-operated mice, expression of FTO and KLF5 was reduced in the GSK3*β* WT MI/R mice; in response to MI/R injury, expression of FTO and KLF5 in GSK3*β* CKO mice was increased compared with GSK3*β* WT mice (Figure 3(e)). Similarly, the phosphorylation level and ubiquitination level of FTO and KLF5 in the H/R-induced cardiomyocytes were increased, while the protein level was decreased (Figures 3(f) and 3(g)).

Next, GSK3*β* was interfered with or overexpressed in cardiomyocytes, and the effects were verified by RT-qPCR. Among them, siGSK3*β*-2 showed better interfering efficacy and was chosen for subsequent experiments (Figure 3(h)). Western blot analysis showed that after GSK3*β* knockdown in cardiomyocytes, phosphorylation of FTO and KLF5 was decreased, and their protein expression was increased, while overexpression of GSK3*β* brought about opposite trends (Figures 3(i) and 3(j)). Further ChIP experiments proved that the ubiquitination levels of WT-FTO and -KLF5 were increased after overexpression of GSK3*β*, while the ubiquitination levels of MUT-FTO (S249A/S253A) and -KLF5 (S292A) did not change significantly (Figures 3(k) and 3(l)). The results of the CHX chase experiment showed that overexpression of GSK3*β* shortened the half-life of FTO and KLF5 (Figure 3(m)).

These results suggested that GSK3*β* promoted FTO and KLF5 ubiquitination degradation by phosphorylation of FTO and KLF5 in cardiomyocytes.

### 3.4. GSK3*β* Promotes H/R-Induced Cardiomyocyte Apoptosis and Oxidative Stress through FTO and KLF5

**Figure 4:**
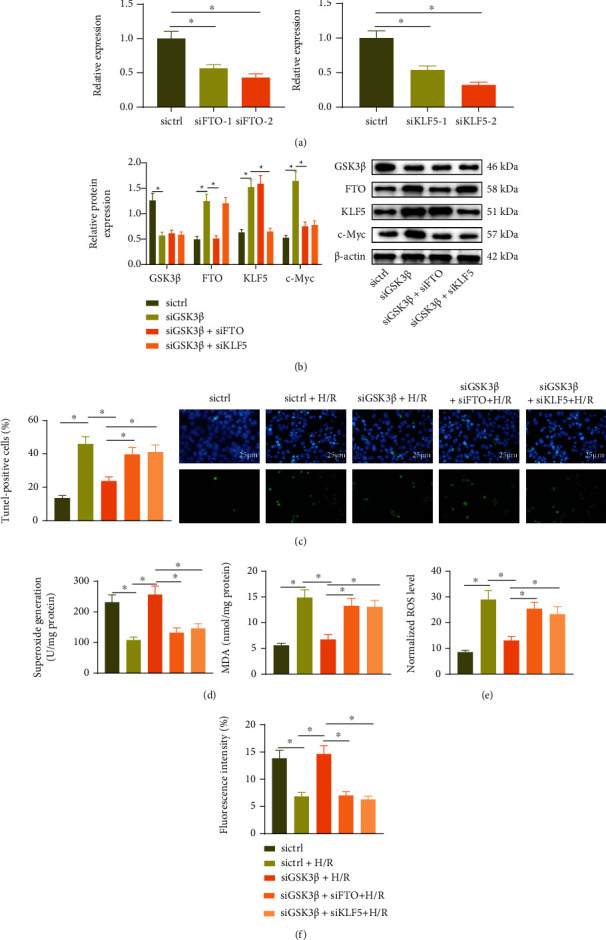
GSK3*β* promotes H/R-induced cardiomyocyte apoptosis and oxidative stress through downregulation of FTO and KLF5: (a) RT-qPCR was used to detect the mRNA level of FTO and KLF5 in cardiomyocytes with siFTO or siKLF5 transfection; (b) Western blot analysis was used to detect the protein of FTO and KLF5 in cardiomyocytes with siFTO or siKLF5 transfection; (c) TUNEL detected the apoptosis of cardiomyocytes with siGSK3*β*, siFTO, or siKLF5 transfection following H/R treatment; the scale bar was 25 *μ*m; (d) the activity of SOD and the content of MDA in cardiomyocytes with siGSK3*β*, siFTO, or siKLF5 transfection following H/R treatment; (e) MitoSOX Red staining fluorescence intensity statistics graph of cardiomyocytes with siGSK3*β*, siFTO, or siKLF5 transfection following H/R treatment; (f) flow cytometry was used to detect JC-1 staining of cells with siGSK3*β*, siFTO, or siKLF5 transfection following H/R treatment. Measurement data were expressed as the mean ± standard deviation. Comparisons among multiple groups were conducted by one-way or repeated measurement ANOVA with Tukey's post hoc test. *p* < 0.05 was indicative of statistical significance. The cell experiment was repeated three times.

We measured the function of GSK3*β* in promoting the ubiquitination and degradation of FTO and KLF5 in MI/R injury. RT-qPCR for transfection efficiency detection of FTO and KLF5 revealed that the interference effect of siFTO-2 and siKLF5-2 was better and thus selected for follow-up experiments (Figure 4(a)). Western blot (Figure 4(b)) exhibited that protein levels of GSK3*β*, FTO, KLF5, and c-Myc reduced in cardiomyocytes treated with the siGSK3*β*. After silencing both GSK3*β* and FTO, the protein levels of GSK3*β* and KLF5 showed no obvious difference, and protein levels of FTO and c-Myc decreased in cardiomyocytes, while after silencing both GSK3*β* and KLF5, the protein levels of GSK3*β* and FTO showed no obvious difference, and protein levels of KLF5 and c-Myc reduced in cardiomyocytes. Then, cardiomyocytes were treated with H/R. Through TUNEL experiment, we found that under H/R injury, the number of apoptotic cells increased; treatment of siGSK3*β* decreased the number of apoptotic cardiomyocytes; the number of apoptotic cardiomyocytes following siGSK3*β*+siFTO+H/R treatment or siGSK3*β*+siKLF5+H/R treatment was increased compared to the siGSK3*β*+H/R treatment (Figure 4(c)). This result indicated that GSK3*β* promoted cardiomyocyte apoptosis by downregulating the expression of FTO and KLF5 during H/R injury.

Next, the activity of SOD and the content of MDA in the cardiomyocytes of each group were tested. The results showed that the SOD activity was decreased and the MDA content was increased after H/R treatment, while siGSK3*β* treatment led to opposite trends. In cells with siGSK3*β*+siFTO+H/R treatment or the siGSK3*β*+siKLF5+H/R treatment, the SOD activity was decreased and the MDA content compared to the siGSK3*β*+H/R treatment (Figure 4(d)). The mitochondrial superoxide probe MitoSOX Red staining showed that the red fluorescence (ROS staining) was increased after H/R treatment which was reduced following siGSK3*β* treatment. Red fluorescence after the siGSK3*β*+siFTO+H/R treatment or siGSK3*β*+siKLF5+H/R treatment was increased compared to the siGSK3*β*+H/R treatment (Figure 4(e)). Moreover, the ratio of red/green fluorescence intensity decreased after H/R treatment which was enhanced after siGSK3*β* treatment. The ratio of red/green fluorescence intensity after the siGSK3*β*+siFTO+H/R treatment or the siGSK3*β*+siKLF5+H/R treatment was decreased relative to the siGSK3*β*+H/R treatment (Figure 4(f)).

Thus, GSK3*β* promoted the oxidative stress of cardiomyocytes by limiting the expression of FTO and KLF5.

### 3.5. FTO and KLF5 Increase the Expression of Myc

**Figure 5:**
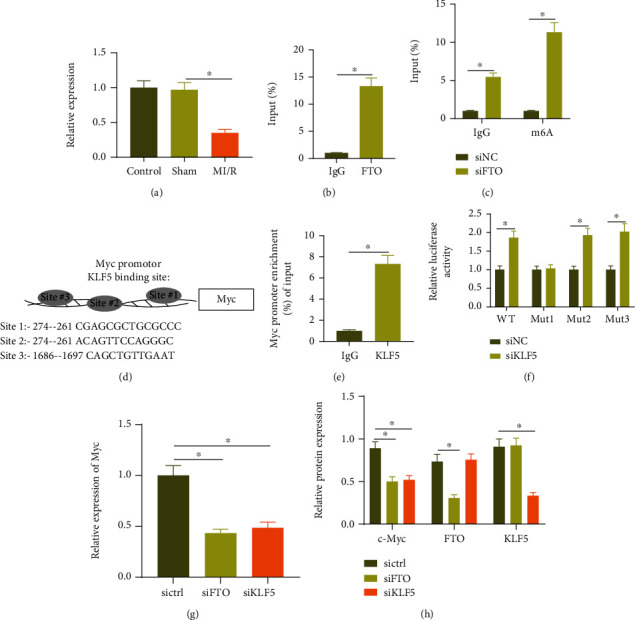
FTO and KLF5 promote Myc expression in cardiomyocytes: (a) RT-qPCR detection of Myc expression in the myocardial tissue of sham-operated mice and MI/R mice; (b) RIP analysis using FTO specific antibody in cardiomyocytes; (c) MeRIP adopted to analyze the m6A modification of Myc mRNA in the cardiomyocytes of each group; (d) bioinformatics analysis of the top three binding sites of KLF5 and Myc promoter; (e) ChIP detected the enrichment of KLF5 in the Myc promoter region in cardiomyocytes; (f) dual-luciferase assay detected the targeting relationship between KLF5 and Myc; (g) RT-PCR was employed to detect the expression of Myc mRNA in the cardiomyocytes of each group; (h) Western blot analysis was used to detect the protein of FTO, KLF5, and Myc in the cardiomyocytes of each group. Measurement data were expressed as the mean ± standard deviation. Data in compliance with normal distribution and homogeneity of variance between two groups were compared using an unpaired *t*-test. Comparisons among multiple groups were conducted by one-way ANOVA or repeated measurement ANOVA with Tukey's post hoc test. *p* < 0.05 was indicative of statistical significance. The cell experiment was repeated three times.

There were literatures showing that FTO promoted Myc expression by removing m6A modification of Myc transcripts, and the combination of KLF5 with the promoter of Myc increased its transcriptional expression [31, 32]. Myc played an important role in MI/R injury [33]. To explore the regulatory relationship between FTO, KLF5, and Myc in MI/R injury, RT-qPCR was operated to detect the expression of Myc in the MI/R mouse model. The results showed that the expression of Myc in the MI/R mouse models was decreased compared with the sham-operated mice (Figure 5(a)). Western blot analysis results showed that the expression of Myc in the H/R-induced cardiomyocytes was decreased (Figure 3(f)). RIP analysis showed that Myc mRNA was significantly enriched in FTO-regulated transcripts (Figure 5(b)). At the same time, the results of MeRIP showed that the m6A modification of Myc mRNA was increased after FTO was knocked down (Figure 5(c)). We also demonstrated reduced Myc mRNA and protein expression after knocking down FTO (Figures 5(g) and 5(h)). These results suggested that FTO promoted the translation and expression of Myc by promoting m6A demethylation of Myc mRNA in cardiomyocytes.

Then, the binding sites of KLF5 and Myc promoter were analyzed through bioinformatics analysis, as shown in Figure 5(d); the top three binding sites were site1: -274–-261, site2: -900–-912, and site3: -1686–-1697. ChIP experiment showed that KLF5 could be largely enriched in the Myc promoter region (Figure 5(e)). In order to confirm the specific binding site, the luciferase report of promoter WT (full-length Myc promoter sequence), promoter MUT-site1 (deleting site1), promoter MUT-site2 (deleting site2), and promoter MUT-site3 (deleting site3) plasmid was constructed, and the experimental results showed that after downregulation of KLF5, the promoter WT group, the promoter MUT-site2 group, and the promoter MUT-site3 group showed an enhanced luciferase signal, but the promoter MUT-site1 group did not change significantly (Figure 5(f)), indicating that the binding site of KLF5 and Myc promoter region was site1. Western blot analysis and RT-qPCR results showed that MYC mRNA and protein were decreased after knocking down KLF5 (Figures 5(g) and 5(h)).

These results suggested that KLF5 and FTO promoted Myc expression.

### 3.6. GSK3*β* Induces MI/R Injury through Myc

**Figure 6:**
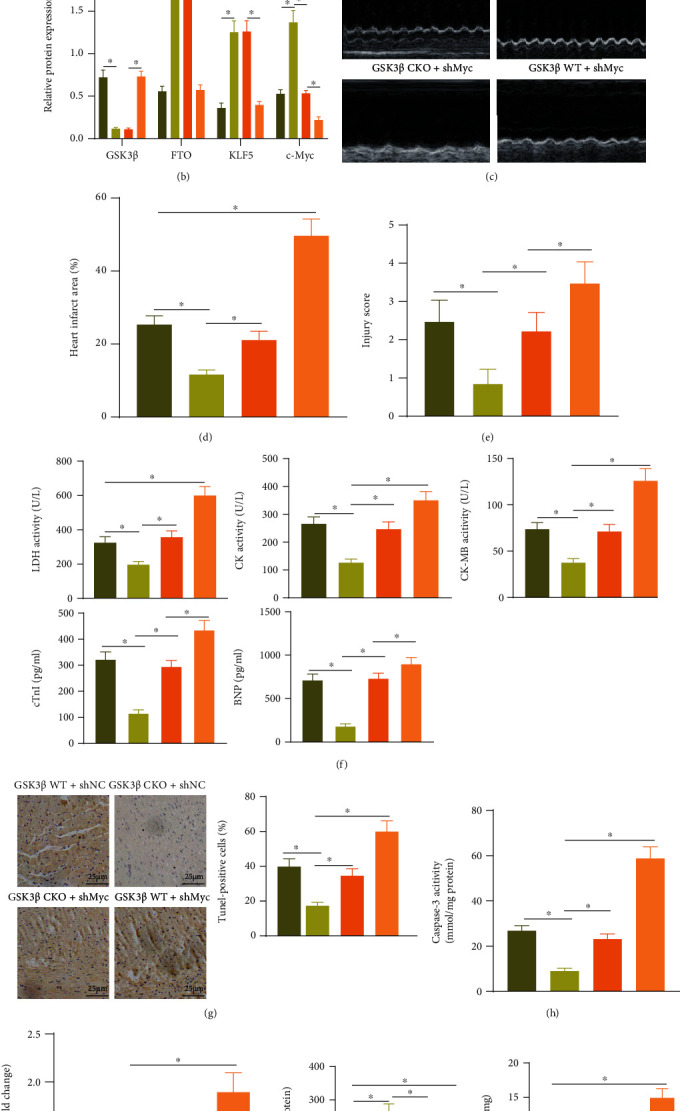
GSK3*β* induces MI/R injury by inhibiting Myc expression: (a) immunohistochemistry was used to detect the expression of Myc in the myocardial tissue of each group of mice; the scale bar was 25 *μ*m; (b) Western blot analysis was used to detect the protein expression of GSK3*β*, FTO, KLF5, and Myc in the myocardial tissue of the different treatment groups; (c) M-mode echocardiographic images of mice in each group; (d) TTC staining statistics of myocardial tissue sections of mice in each group; (e) HE staining diagram of myocardial tissue of each group of mice; (f) a statistical diagram of the expression of LDH, CK, and CK-MB in the peripheral blood of each group of mice; (g) TUNEL detected the apoptosis of cardiomyocytes in the myocardial tissue of each group of mice; the scale bar was 25 *μ*m; (h) detection of the activity of Caspase-3 in the myocardial tissue of each group of mice; (i) DCF-DA staining of myocardial tissue sections in each group of mice was used to detect ROS in cells; (j) the activity of SOD and the content of MDA in the hearts of mice in each group. *N* = 8. Measurement data were expressed as the mean ± standard deviation. Comparisons among multiple groups were conducted by one-way ANOVA or repeated measurement ANOVA with Tukey's post hoc test. *p* < 0.05 was indicative of statistical significance.

We then verified the above findings *in vivo*. Immunohistochemistry showed that, without knocking down GSK3*β*, compared with the sham-operated mice, the expression of Myc in the MI/R mice was reduced. In response to MI/R injury, compared with the GSK3*β* WT treatment, the expression of Myc after the GSK3*β* CKO was increased (Figure 6(a)). Next, Myc was knocked down in the hearts of mice in each group, and Western blot analysis results showed that there was no significant change in the protein levels of GSK3*β*, FTO, and KLF5, but the Myc protein level was decreased after the GSK3*β* CKO+shMyc treatment relative to the GSK3*β* CKO+shNC treatment as well as after the GSK3*β* WT+shMyc treatment relative to the GSK3*β* WT+shNC treatment (Figure 6(b)).

After MI/R treatment, echocardiography was performed. The results showed that compared with GSK3*β* CKO+shNC treatment, LVEDD, LVESD, and LVEDP were increased, while LVEF, LVFS, and LVSP were decreased after GSK3*β* CKO+shMyc treatment, and when compared with GSK3*β* WT+shNC treatment, GSK3*β* WT+shMyc treatment showed the same trends (Figure 6(c), Supplementary Table 5). TTC and HE staining indicated that the area of myocardial infarction was increased and the pathological changes were aggravated with elevated pathological scores in the GSK3*β* CKO+shMyc treatment and the GSK3*β* WT+shMyc treatment compared with the GSK3*β* CKO+shNC treatment and the GSK3*β* WT+shNC treatment, respectively (Figures 6(d) and 6(e), Supplementary Figure 1C). Then, we also found that expression of LDH, CK, CK-MB, cTnI, and BNP after GSK3*β* CKO+shMyc treatment or the GSK3*β* WT+shMyc treatment was increased relative to the GSK3*β* CKO+shNC treatment or GSK3*β* WT+shNC treatment, respectively (Figure 6(f)). Moreover, apoptosis of cardiomyocytes was increased with elevated Caspase-3 expression under GSK3*β* CKO+shMyc treatment compared to the GSK3*β* CKO+shNC treatment and also enhanced following the GSK3*β* WT+shMyc treatment compared to the GSK3*β* WT+shNC treatment (Figures 6(g) and 6(h)).

To explore the effect of GSK3*β* regulating Myc on oxidative stress, the ROS fluorescent probe DCFH-DA was used to stain the myocardial tissue sections of mice in each group. It was found that ROS after the GSK3*β* CKO+shMyc treatment was increased relative to the GSK3*β* CKO+shNC treatment and also enhanced after the GSK3*β* WT+shMyc treatment relative to the GSK3*β* WT+shNC treatment (Figure 6(i)). In addition, compared to the GSK3*β* CKO+shNC treatment or the GSK3*β* WT+shNC treatment, the SOD activity was decreased and the MDA content was increased after the GSK3*β* CKO+shMyc treatment or the GSK3*β* WT+shMyc treatment (Figure 6(j)).

These results suggested that GSK3*β*, as upstream of FTO and KLF5, regulated Myc expression to promote MI/R injury, and knockdown of GSK3*β* could effectively alleviate MI/R injury.

## 4. Discussion

MI/R injury is the main cause of coronary artery disease-related morbidity and mortality [34]. Our results demonstrated that in MI/R injury, innate immunity and subsequent inflammation bear great responsibility in the expansion of myocardial injury [35]. MI/R injury often induces oxidative stress and inflammation, leading to myocardial cell apoptosis and necrosis [36]. Herein, this study mainly explored the involvement of GSK3*β*/FTO/KLF5/Myc on MI/R injury and underlying molecular mechanisms associated with cardiomyocyte apoptosis and oxidative stress. The available well-established evidence revealed that GSK3*β* caused downregulation of FTO and KLF5 induced MI/R injury by inhibition of Myc coupling with stimulated cardiomyocyte apoptosis and oxidative stress.

To start with, the MI/R mouse model and H/R cell model were established with elevated GSK3*β* found. More specifically, GSK3*β* increased cardiomyocyte apoptosis and oxidative stress, thereby aggravating the MI/R injury. GSK-3*β*, the main constituent of the RISK pathway, is a critical mediator of survival in cardiac myocytes, thus functioning in the pathogenesis of MI/R injury [37]. The phosphorylation found within the amino-terminal domain of GSK-3*β* at Ser9 leads to limited GSK-3 kinase activity, highlighting that inactivation of GSK-3*β* is cardioprotective [38, 39]. Moreover, GSK3*β* mediates cardiomyocyte apoptosis in response to high glucose [40]. After sevoflurane preconditioning in mice with MI/R injury, GSK3*β* inhibits the cardioprotective effects associated with the recovery of mitochondrial function and the inhibition of endoplasmic reticulum stress [41]. Similarly, a corroborating study has previously suggested that GSK3*β* is known as a hormone control regulator that maintains glucose stability, which exacerbates cardiac MI/R injury [25]. These findings support that GSK3*β* enhanced cardiomyocyte apoptosis and oxidative stress induced by MI/R injury.

Additionally, the finding in the current study demonstrated that GSK3*β* promoted ubiquitination and degradation of FTO and KLF5 to regulate cardiomyocyte apoptosis and oxidative stress. Protein ubiquitination plays an important role in the processes of cell degradation, DNA repair, endocytosis, and inflammation [42]. FTO, a key demethylase involved in a variety of physiological processes, regulates mA demethylation to act importantly in cerebral ischemia-reperfusion injury [12, 43]. KLF5 is capable of modulating cell differentiation, proliferation, migration, and apoptosis as well as cardiovascular remodeling [44]. KLF5 can attenuate cardiomyocyte inflammation induced by oxygen-glucose deprivation/reperfusion [14]. In addition, KLF5 contributes to oxidative stress and diabetic cardiomyopathy [45]. Importantly, GSK3*β* accelerates the ubiquitination degradation of FTO and KLF5 through phosphorylation of FTO (phosphorylation sites S249 and S253) and KLF5 (human site S292 and mouse site S292) [10, 30]. All above indicated that FTO and KLF5 facilitate cardiomyocyte apoptosis and oxidative stress to increase MI/R injury.

Furthermore, we further confirmed that FTO and KLF5 could induce the elevation of Myc expression. Myc has been validated to be dispensable for the development of cardiomyocytes, which is conducive to heart development [46]. High expression of Myc has also been detected in cells with inhibited ischemia-induced oxidative stress and apoptosis [47]. Moreover, upregulated Myc expression is involved in ameliorating MI/R injury [33]. FTO promoted MYC expression by removing m6A modification of MYC transcripts, and the combination of KLF5 with the promoter of Myc increased its transcriptional expression [31, 32]. Our study also proved that Myc silencing contributed to stimulated cardiomyocyte apoptosis and oxidative stress, thus enhancing MI/R injury.

## 5. Conclusions

**Figure 7:**
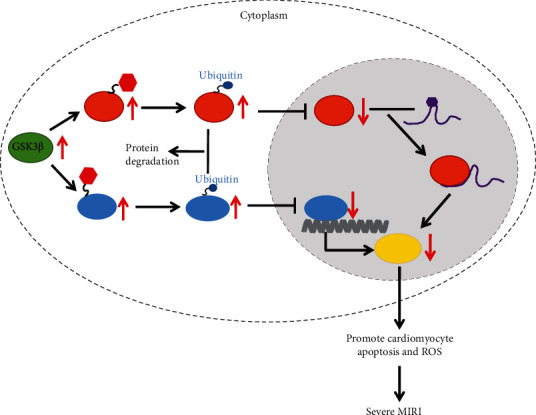
Schematic map of GSK3*β*-mediated FTO and KLF5 in MI/R injury. In the case of MI/R injury, the expression of GSK3*β* in cardiomyocytes was increased, which promoted the phosphorylation of FTO and KLF5, thereby promoting their ubiquitination and degradation. The low expression of FTO promoted the m6A modification of Myc mRNA and resulted in the inhibition of Myc translation, while the low expression of KLF5 inhibited the transcription expression of Myc. Therefore, the expression of Myc in cardiomyocytes was reduced, leading to MI/R-induced cardiomyocyte apoptosis and increased ROS, thereby exacerbating MI/R injury.

In conclusion, our study collectively showed that GSK3*β* could enhance phosphorylation of FTO and KLF5 to downregulate Myc, thus inducing cardiomyocyte apoptosis and oxidative stress in MI/R injury (Figure 7). On contrary, deletion of GSK3*β* could attenuate MI/R injury. The present study lays the groundwork for the development of new therapeutic targets for MI/R injury. However, the specific mechanism of how Myc leads to suppressed cardiomyocyte apoptosis and oxidative stress is still unknown. Therefore, more details remain to be elucidated in future investigations.

## Data Availability

All data generated or analyzed during this study are included in this published article.

## Abbreviations

MI/R: Myocardial ischemia/reperfusion
GSK3: Glycogen synthase kinase-3
GSK-3*β*: Glycogen synthase kinase 3*β*
m6A: N-Methyladenosine
FTO: Fat mass and obesity-associated enzymes
KLF5: Kruppel-like zinc-finger transcription factor 5
GEO: Gene Expression Omnibus
NCBI: National Center for Biotechnology Information
UCSC: University of California Santa Cruz
SPF: Specific-pathogen-free
shRNA: Short hairpin RNA
LVEDD: Left ventricular end diastolic dimension
LVESD: Left ventricular end systolic diameter
LVEF: Left ventricular ejection fraction
LVFS: Left ventricular fraction shortening
LVSP: Left ventricular systolic pressure
LVEDP: Left ventricular end diastolic pressure
MBs: Myocardial enzymes
HE: Hematoxylin and eosin
TTC: Triphenyltetrazolium chloride
IS: Infarct size
DCFDA: 2′,7′-Dichlorodihydrofluorescein diacetate
SOD: Superoxide dismutase
MDA: Malondialdehyde
PBS: Phosphate-buffered saline
DMEM: Dulbecco's modified Eagle medium
siRNAs: Short interfering RNAs
NCs: Negative controls
ROS: Reactive oxygen species
qRT-PCR: Reverse transcription quantitative polymerase chain reaction
GAPDH: Glyceraldehyde-3-phosphate dehydrogenase
RIPA: Radioimmunoprecipitation assay
BCA: Bicinchoninic acid
SDS: Sodium dodecyl sulfate
BSA: Bovine serum albumin
IgG: Immunoglobulin G
HRP: Horseradish peroxidase
DAB: Diaminobenzidine
RIP: RNA immunoprecipitation
TDT: Terminal deoxynucleotidyl transferase
TUNEL: Terminal deoxynucleotidyl transferase-mediated dUTP-biotin nick end-labeling
ChIP: Chromatin immunoprecipitation
ANOVA: Analysis of variance.

## Supplementary Materials

Supplementary Materials Supplementary Figure 1: (A) Representative HE staining of Figure 1(c) (the arrow points to the obvious lesion); (b) representative HE staining of Figure 2(d) (the arrow points to the obvious lesion); (c) representative HE staining of Figure 6(e) (the arrow points to the obvious lesion). Supplementary Table 1: siRNA sequences. Note: siRNAs: short interfering RNAs; GSK3*β*: glycogen synthase kinase-3; FTO: fat mass and obesity-associated enzymes; KLF5: Kruppel-like zinc-finger transcription factor 5; sictrl: short interfering RNAs against negative controls. Supplementary Table 2: primer sequences for RT-qPCR. Note: RT-qPCR: reverse transcription quantitative polymerase chain reaction; GSK3*β*: glycogen synthase kinase-3; FTO: fat mass and obesity-associated enzymes; KLF5: Kruppel-like zinc-finger transcription factor 5; GAPDH: glyceraldehyde-3-phosphate dehydrogenase; F: forward; R: reverse. Supplementary Table 3: comparison of the results of echocardiography in the control, sham, and MI/R groups. Note: LVEDD: left ventricular end diastolic dimension; LVESD: left ventricular end systolic diameter; LVEF: left ventricular ejection fraction; LVFS: left ventricular fraction shortening; LVSP: left ventricular systolic pressure; LVEDP: left ventricular end diastolic pressure; MI/R: myocardial ischemia/reperfusion. ^∗^*p* < 0.05 vs. sham-operated mice. Supplementary Table 4: comparison of the results of echocardiography in GSK3*β* WT and GSK3*β* CKO mice following MI/R. Note: LVEDD: left ventricular end diastolic dimension; LVESD: left ventricular end systolic diameter; LVEF: left ventricular ejection fraction; LVFS: left ventricular fraction shortening; LVSP: left ventricular systolic pressure; LVEDP: left ventricular end diastolic pressure; MI/R: myocardial ischemia/reperfusion. ^∗^*p* < 0.05 vs. cells treated with I/R+GSK3*β* WT. Supplementary Table 5: comparison of the results of echocardiography in GSK3*β* WT and GSK3*β* CKO mice with shMyc transfection. Notes: LVEDD: left ventricular end diastolic dimension; LVESD: left ventricular end systolic diameter; LVEF: left ventricular ejection fraction; LVFS: left ventricular fraction shortening; LVSP: left ventricular systolic pressure; LVEDP: left ventricular end diastolic pressure; MI/R: myocardial ischemia/reperfusion. ^∗^*p* < 0.05 vs. cells treated with GSK3*β* CKO+shNC; ^#^*p* < 0.05 vs. cells treated with GSK3*β* WT+shNC.
