# Supplementary material for: GSK3β Exacerbates Myocardial Ischemia/Reperfusion Injury by Inhibiting Myc
Source: Oxid Med Cell Longev. 2022 Apr 29;2022:2588891. doi: 10.1155/2022/2588891 (PMC9076327; doi:10.1155/2022/2588891)
Supplement: Supplementary Materials — Supplementary Figure 1: (A) Representative HE staining of Figure 1(c) (the arrow points to the obvious lesion); (b) representative HE staining of Figure 2(d) (the arrow points to the obvious lesion); (c) representative HE staining of Figure 6(e) (the arrow points to the obvious lesion). Supplementary Table 1: siRNA sequences. Note: siRNAs: short interfering RNAs; GSK3β: glycogen synthase kinase-3; FTO: fat mass and obesity-associated enzymes; KLF5: Kruppel-like zinc-finger transcription factor 5; sictrl: short interfering RNAs against negative controls. Supplementary Table 2: primer sequences for RT-qPCR. Note: RT-qPCR: reverse transcription quantitative polymerase chain reaction; GSK3β: glycogen synthase kinase-3; FTO: fat mass and obesity-associated enzymes; KLF5: Kruppel-like zinc-finger transcription factor 5; GAPDH: glyceraldehyde-3-phosphate dehydrogenase; F: forward; R: reverse. Supplementary Table 3: comparison of the results of echocardiography in the control, sham, and MI/R groups. Note: LVEDD: left ventricular end diastolic dimension; LVESD: left ventricular end systolic diameter; LVEF: left ventricular ejection fraction; LVFS: left ventricular fraction shortening; LVSP: left ventricular systolic pressure; LVEDP: left ventricular end diastolic pressure; MI/R: myocardial ischemia/reperfusion. ∗p < 0.05 vs. sham-operated mice. Supplementary Table 4: comparison of the results of echocardiography in GSK3β WT and GSK3β CKO mice following MI/R. Note: LVEDD: left ventricular end diastolic dimension; LVESD: left ventricular end systolic diameter; LVEF: left ventricular ejection fraction; LVFS: left ventricular fraction shortening; LVSP: left ventricular systolic pressure; LVEDP: left ventricular end diastolic pressure; MI/R: myocardial ischemia/reperfusion. ∗p < 0.05 vs. cells treated with I/R+GSK3β WT. Supplementary Table 5: comparison of the results of echocardiography in GSK3β WT and GSK3β CKO mice with shMyc transfection. Notes: LVEDD: left ventricular end di [file 2588891.f1.docx]

**
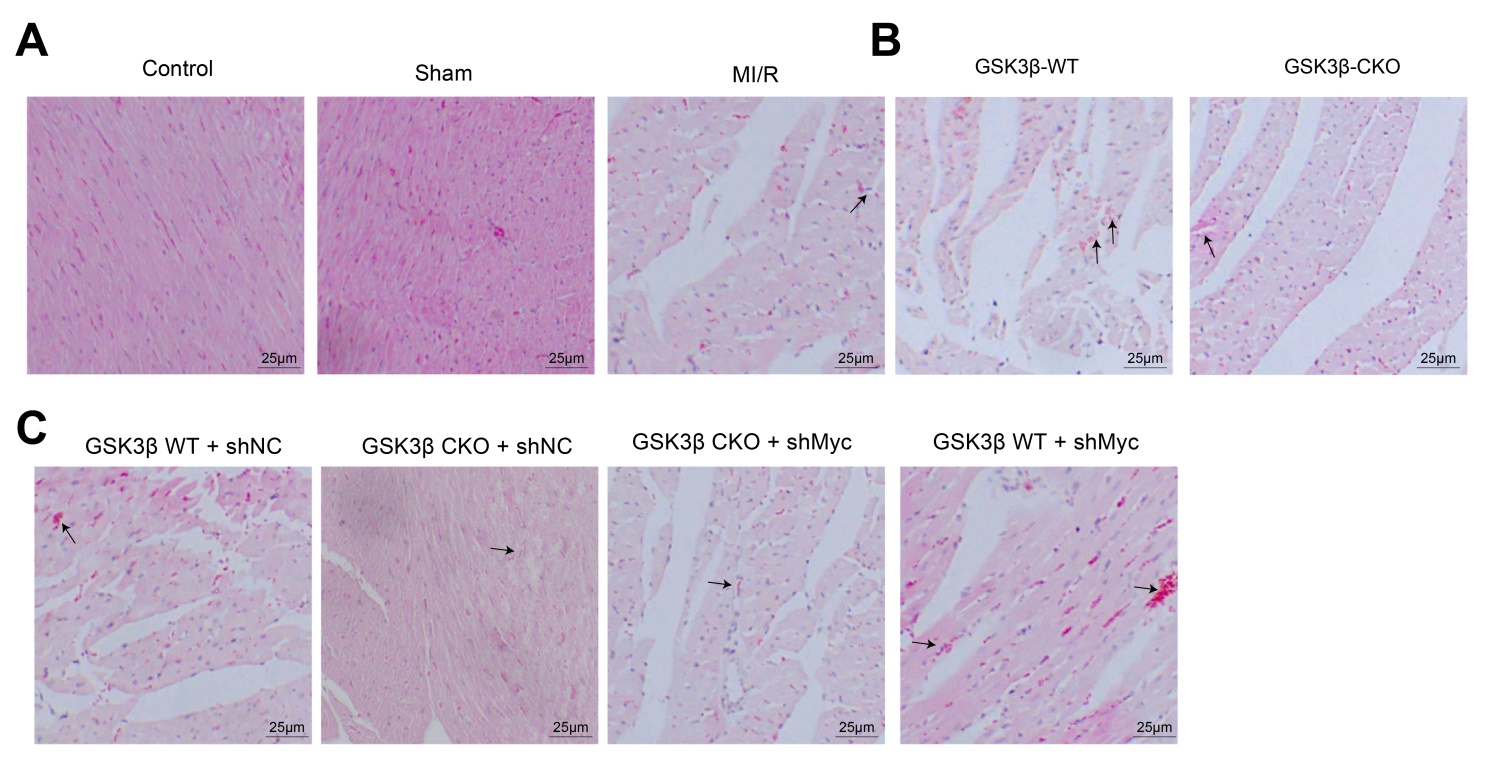
**

**Supplementary Figure 1** A, Representative HE staining of figure 1C (The arrow points to the obvious lesion); B, Representative HE staining of Figure 2D (The arrow points to the obvious lesion); C, Representative HE staining of Figure 6E (The arrow points to the obvious lesion).

**Supplementary Table 1** siRNAs sequences

| Name | Primer sequence (5’-3’) |
| --- | --- |
| siGSK3β-1 | CCTTAACCTGGTGCTGGACTATGTT |
| siGSK3β-2 | GCTTCTGCATCAGCTTCCAACTCCA |
| siFTO-1 | GAGCAGCCTACAACGTGACTTTGCT |
| siFTO-2 | GAGTGCTCAACAGGCACCTTGGATT |
| siKLF5-1 | GCCTCAGTGGTAGACCAGTTCTTCA |
| siKLF5-2 | CAGACGGCAGTAATGGACACCCTTA |
| sictrl | GGTGAAGGTCGGAGTCAACG |

### Note: siRNAs, short interfering RNAs; GSK3β, glycogen synthase kinase-3; FTO, fat mass and obesity-associated enzymes; KLF5, Kruppel-like zinc-finger transcription factor 5; sictrl, short interfering RNAs against negative controls.

**Supplementary Table 2** Primer sequences for RT-qPCR

| Genes | Primer sequence (5’-3’) |
| --- | --- |
| GSK3β | F:5’-ATCGCCACATGCAGTCACA-3’ |
|  | R:5’-AGCCACACCAAACCATGGAC-3’ |
| FTO | F:5’-AGCCCCTGGGTCTCCATAAT-3’ |
|  | R:5’-ATGGTGACAGGGAGCAACAG-3’ |
| KLF5 | F:5’-GGACTCATACGGGCGAGAAG-3’ |
|  | R:5’-TAAAGGATGGCAGAGCGGAC-3’ |
| Myc | F:5’-TCTCTCCTTCCTCGGACTCG-3’ |
|  | R:5’-GTGTCTCCTCATGCAGCACT-3’ |
| GAPDH | F:5’-CAGGTTGTCTCCTGCGACTT-3’ |
|  | R:5’-TATGGGGGTCTGGGATGGAA-3’ |

### Note: RT-qPCR, reverse transcription quantitative polymerase chain reaction; GSK3β, glycogen synthase kinase-3; FTO, fat mass and obesity-associated enzymes; KLF5, Kruppel-like zinc-finger transcription factor 5; GAPDH, glyceraldehyde-3-phosphate dehydrogenase; F, forward; R, reverse.

**Supplementary Table 3** Comparison of the results of echocardiography in control, sham, MI/R groups

| Group/Parameter | LVEDD | LVESD | LVEF(%) | LVFS(%) | LVSP | LVEDP |
| --- | --- | --- | --- | --- | --- | --- |
|  | (mm) | (mm) |  |  | (mm Hg) | (mm Hg) |
| control | 4.31±0.39 | 2.64±0.37 | 81.52±7.33 | 53.02±5.48 | 152.31±17.63 | 6.15±1.43 |
| Sham | 4.03±0.52 | 2.21±0.34 | 76.22±7.89 | 47.31±4.06 | 143.56±19.64 | 5.85±1.24 |
| MI/R | 7.03±1.01* | 5.33±0.62* | 36.85±3.47* | 27.59±2.48* | 61.53±5.84* | 12.07±2.03* |

Note: LVEDD: the left ventricular end diastolic dimension; LVESD: left ventricular end systolic diameter; LVEF: left ventricular ejection fraction; LVFS: left ventricular fraction shortening; LVSP: left ventricular systolic pressure; LVEDP: left ventricular end diastolic pressure; MI/R: myocardial ischemia/reperfusion. * *p* < 0.05 vs Sham-operated mice

**Supplementary Table 4** Comparison of the results of echocardiography in GSK3β WT and GSK3β CKO mice following MI/R

| Group/Parameter | LVEDD | LVESD | LVEF(%) | LVFS(%) | LVSP | LVEDP |
| --- | --- | --- | --- | --- | --- | --- |
|  | (mm) | (mm) |  |  | (mm Hg) | (mm Hg) |
| MI/R + GSK3β WT | 8.03±0.96 | 5.32±0.47 | 41.96±5.37 | 29.64±3.45 | 61.08±5.38 | 12.99±1.76 |
| MI/R + GSK3β CKO | 4.03±0.52* | 2.37±0.41* | 83.65±7.02* | 53.18±5.44* | 125.63±8.59* | 4.71±0.68* |

Note: LVEDD: the left ventricular end diastolic dimension; LVESD: left ventricular end systolic diameter; LVEF: left ventricular ejection fraction; LVFS: left ventricular fraction shortening; LVSP: left ventricular systolic pressure; LVEDP: left ventricular end diastolic pressure; MI/R: myocardial ischemia/reperfusion. * *p* < 0.05 vs cells treated with I/R + GSK3β WT

**Supplementary Table 5** Comparison of the results of echocardiography in GSK3β WT and GSK3β CKO mice with shMyc transfection

| Group\Parameter | LVEDD | LVESD | LVEF(%) | LVFS(%) | LVSP | LVEDP |
| --- | --- | --- | --- | --- | --- | --- |
|  | (mm) | (mm) |  |  | (mm Hg) | (mm Hg) |
| GSK3βWT + shNC | 8.06±0.76 | 4.98±0.51 | 45.73±4.81 | 29.34±3.05 | 61.33±4.07 | 13.06±1.89 |
| GSK3β CKO + shNC | 3.56±0.48 | 3.01±0.45 | 86.37±6.03 | 50.73±4.88 | 124.63±6.03 | 6.01±0.85 |
| GSK3β CKO + shMyc | 6.33±0.77* | 6.96±0.59* | 43.77±4.26* | 26.85±2.74 | 26.86±3.12* | 14.51±2.54* |
| GSK3β WT + shMyc | 13.56±2.47# | 9.31±0.86# | 19.67±1.77# | 13.25±2.48# | 31.48±2.57# | 26.33±3.54# |

Notes: LVEDD: the left ventricular end diastolic dimension; LVESD: left ventricular end systolic diameter; LVEF: left ventricular ejection fraction; LVFS: left ventricular fraction shortening; LVSP: left ventricular systolic pressure; LVEDP: left ventricular end diastolic pressure; MI/R: myocardial ischemia/reperfusion. **p* < 0.05 vs cells treated with GSK3β CKO + shNC; # *p* < 0.05 vs cells treated with GSK3β WT + shNC.
